# Positive Nursing Practice Environment: A Concept Analysis

**DOI:** 10.3390/nursrep14040222

**Published:** 2024-10-17

**Authors:** Soraia Pereira, Marlene Ribeiro, Mariana Mendes, Rosilene Ferreira, Eduardo Santos, Cintia Fassarella, Olga Ribeiro

**Affiliations:** 1 Abel Salazar Biomedical Sciences Institute, University of Porto, 4050-313 Porto, Portugal; marlene.ribeiro@chts.min-saude.pt; 2 Northern Health School of the Portuguese Red Cross, 3720-126 Oliveira de Azeméis, Portugal; 3 Center for Health Technology and Services Research (CINTESIS@RISE), 4200-450 Porto, Portugal; esantos@essv.ipv.pt (E.S.); olgaribeiro@esenf.pt (O.R.); 4 Tâmega and Sousa Local Health Unit, 4560-136 Penafiel, Portugal; 5 Postgraduate Program in Nursing, Federal University of Santa Catarina, Florianópolis 88040-900, Brazil; mariana.mendes@unochapeco.edu.br; 6 Faculty of Nursing, The State University of Rio de Janeiro, Rio de Janeiro 20031-040, Brazil; ferreira.rosilene@uerj.br (R.F.); cintia.silva.fassarella@uerj.br (C.F.); 7 Polytechnic Institute of Viseu, Higher School of Health, 3500-843 Viseu, Portugal; 8 Health Sciences Research Unit-Nursing (UICISA: E), 3045-043 Coimbra, Portugal; 9 Evidence-Based Practice Center of Portugal (PCEBP): A JBI Centre of Excellence, 3045-043 Coimbra, Portugal; 10 Nursing School of Porto, 4200-072 Porto, Portugal

**Keywords:** nursing, work environment, working conditions, concept analysis

## Abstract

**Background/Objectives:** In recent years, research has increasingly highlighted the significance of nursing practice environments, linking positive settings with enhanced job satisfaction, professional autonomy, and care quality. Such environments can decrease turnover, stress, and costs while improving patient safety. Despite this extensive literature, there is limited consensus on defining a ‘positive nursing practice environment’, highlighting the need for a systematic analysis to advance understanding and application. This study aims to explore and clarify the concept of a ‘Positive Nursing Practice Environment’. **Methods:** This study applied Walker and Avant’s approach for concept analysis, conducting a comprehensive database search to gather relevant evidence. To review the available evidence on the concept of nursing practice environments, we followed the methodology proposed by JBI for scoping reviews. **Results:** The inclusion of 166 studies meeting the criteria provided a broad understanding of the topic, revealing 10 key attributes of a ‘positive nursing practice environment’ and identifying various antecedents and consequences affecting clients, nurses, and institutions. **Conclusions:** The concept analysis of a ‘Positive Nursing Practice Environment’ offers valuable insights into nurses’ working conditions, systematically identifying characteristics that impact professionals, patients, and institutions. This analysis lays the groundwork for future research and practical improvements in nursing practice environments. This study was not registered.

## 1. Introduction

Over the past decade, there has been growing interest in researching nursing practice environments. Numerous studies have underscored the benefits of investing in the quality of these environments [1,2]. Research has consistently shown a connection between favorable and healthy nursing practice environments and improved professional satisfaction, safe practice conditions, autonomy, psychological and physical well-being, professional retention, and higher quality of care [1,3,4]. 

Moreover, the literature highlights additional benefits, such as reduced turnover, enhanced financial viability due to lower institutional costs, decreased insecurity among professionals and patients, and a reduction in stress, fatigue, and burnout [2,5,6]. This triple impact—on clients, professionals, and institutions—has spurred the development of various global reports and initiatives emphasizing the importance of nursing work environments as a catalyst for quality healthcare [7,8].

The Institute of Medicine (IOM) report established a significant correlation between nurse practice environments and patient safety, emphasizing the need for active nursing leadership, appropriate nurse-patient ratios, and the inclusion of nurses in organizational management [7]. Similarly, in 2021, the World Health Organization (WHO) recognized the critical role of nursing work environments, underscoring their link to patient safety [8]. 

To drive organizational innovation, the Magnet Recognition Program was introduced in the 1990s by the American Nurses Credentialing Center (ANCC) [9]. This voluntary program recognizes hospitals with exceptional nursing work environments and high-quality customer service [10]. Evidence of the program’s effectiveness has been well documented, showing improvements in job satisfaction, reduced burnout, and lower turnover intentions among nursing professionals [9,11]. Additionally, it has positively impacted patient outcomes, including reduced mortality and shorter hospital stays, as well as institutional performance indicators such as cost reduction [12,13].

In 2001, the American Association of Critical-Care Nurses (AACN) committed to developing healthy work environments for nurses caring for critically ill patients. According to the AACN, key elements for maintaining such environments include skilled communication, true collaboration, effective decision-making, appropriate staffing, meaningful recognition, and authentic leadership [14].

Since then, various definitions and terms related to nursing practice environments have emerged in the literature. For example, Lake (2002) defined the nursing practice environment as “*the set of characteristics of the work context that facilitate or constrain professional nursing practice*” [15]. In 2007, the International Council of Nurses (ICN) described a positive practice environment as one that supports excellence and decent work [16]. Specifically, ICN emphasized environments that ensure staff health and safety, support high-quality patient care, and foster motivation and productivity. Similarly, the Registered Nurses’ Association of Ontario (RNAO) defined a healthy work environment as a practice setting that maximizes the well-being of nurses, the quality of patient outcomes, and organizational performance [17]. In 2018, the American Nurses Association (ANA) defined a healthy work environment for nurses as “*a safe, empowering, and satisfying workplace*” [18].

Despite the extensive discussion of nursing practice environments, our comprehensive literature review revealed only one study that established a consensus on defining a positive work environment for healthcare professionals through a literature review and a three-round Delphi study [19]. This gap in the literature suggests an opportunity to further explore and systematize existing information on the concept of a ‘Positive Nursing Practice Environment’.

The purpose of this study is to conduct a concept analysis of the ‘Positive Nursing Practice Environment’ using the Walker and Avant framework [20]. By identifying defining attributes, models, contrary cases, antecedents, consequences, and empirical referents, we aim to enhance the understanding of this concept and contribute to the development of a theoretical definition and measurement tool.

## 2. Materials and Methods

This study employed Walker and Avant’s approach for concept analysis [20], conducting a comprehensive search of databases to gather relevant evidence on the topic. The concept analysis approach consists of eight stages: selecting a concept, determining the purpose of the analysis, identifying all uses of the concept, defining attributes, identifying a model case, identifying borderline, related, and contrary cases, identifying antecedents and consequences, and defining empirical referents [20].

### 2.1. Concept Analysis Method

Concept analysis involves a detailed examination of a word or phrase to understand its meaning and its role in theory development. The goal is to distinguish a specific concept from others that may be similar but different in key aspects. This process clarifies the concept, develops a theoretical definition, and provides tools for future research [20]. In the context of healthcare, analyzing the concept of a “Positive Nursing Practice Environment” can enhance understanding of its characteristics and elements, potentially leading to the creation of environments that promote quality and safety in nursing practice.

### 2.2. Data Sources

To review available evidence on the concept of nursing practice environments, we used the methodology proposed by JBI for scoping reviews and adhered to PRISMA-ScR guidelines to maintain the rigor and detail we aim to incorporate in this concept analysis [21]. Scoping reviews are particularly valuable for mapping key concepts underpinning a topic, clarifying concepts and definitions in the literature, and identifying key characteristics or factors related to a concept [21]. This has proven to be an effective tool for conducting a comprehensive and detailed analysis of the concept of a positive nursing practice environment.

Eligibility criteria were defined using the PCC framework [22]. Only studies focusing on nurses (Population) were included to maintain the relevance of the findings. The studies considered had to address the concept of the nursing practice environment (Concept) within any clinical nursing context (Context).

The research strategy consisted of three phases. First, we conducted an initial limited search of MEDLINE (PubMed) and CINAHL (EBSCO) to identify relevant articles. The text words and index terms from these articles were then used to refine and develop a comprehensive search strategy, which is outlined in Table 1. In the second phase, using the refined keywords and index terms, we searched the following databases: MEDLINE (PubMed), CINAHL (EBSCO), Mediclatina (EBSCO), Portugal’s Open Access Scientific Repository (RCAAP), and WorldCat. We included both published studies and gray literature from 2007 onwards, as the concept of a positive practice environment was first introduced by the ICN in that year [16]. Only peer-reviewed journal articles were considered, excluding opinion pieces, editorials, and methodological studies focused solely on evaluating psychometric properties of instruments. In this final phase, we examined the reference lists of included studies for any additional relevant studies but found none.

Two independent reviewers (SP and MR) evaluated the titles and abstracts based on predefined inclusion criteria. Studies that met these criteria were read in full. In cases of disagreement between reviewers, a third reviewer (MM) facilitated a consensus through discussion. Data from selected studies were extracted using a standardized data extraction tool developed by the review team. This process included specific details regarding antecedents, attributes, consequences, and instruments used to evaluate the concept. Two independent reviewers (SP and OR) performed the data extraction. No contact with the authors of the included studies was necessary for further information or clarification.

## 3. Results

**Figure 1:**
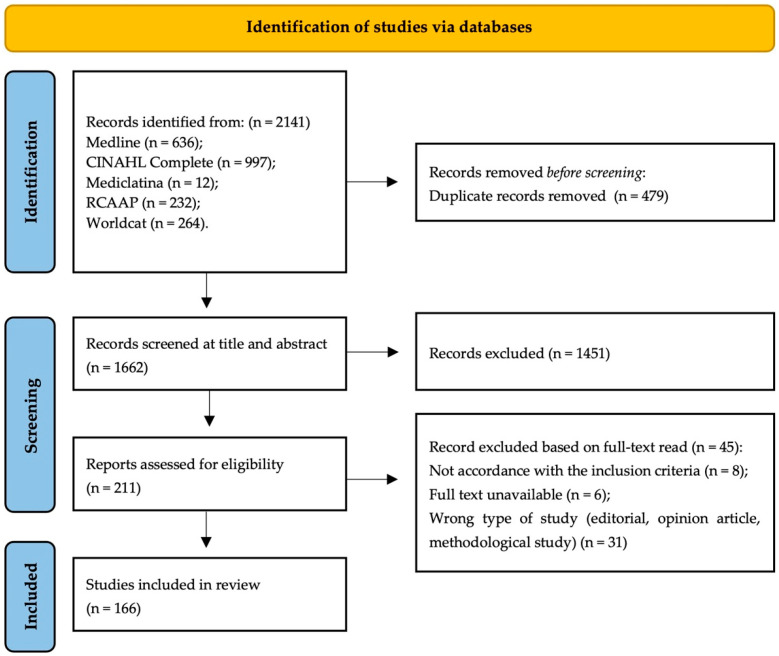
PRISMA-ScR flowchart. Schematic presentation of the flow of information through the different stages of the review, with the number of records identified, included, and excluded and the respective reasons for exclusions.

A total of 2141 studies were identified through searches in the various databases mentioned above. After eliminating duplicates, 1662 studies remained. Following the review process, 166 studies met the inclusion criteria and were included in this analysis (Figure 1—PRISMA-ScR flowchart). The majority of the included studies are journal articles (n = 146), primarily cross-sectional (n = 55), descriptive (n = 44), reviews (n = 24), and qualitative studies (n = 14). Additionally, 20 dissertations and theses were included from gray literature. Most studies were conducted in hospital settings (n = 115), and around 120 of the included studies were published between 2018 and 2023. Due to the large number of included studies, only the most recent citations supporting the review’s findings are highlighted. However, all included studies are available in the Supplementary Materials.

### 3.1. Uses of the Concept

The concept of ‘Positive Nursing Practice Environment’ was applied across diverse settings in the literature, including hospitals, primary healthcare centers, nursing homes, prisons, private clinics, and schools, among others. This broad applicability suggests that the concept is relevant to any setting where nursing practice is conducted.

### 3.2. Defining Attributes

**Figure 2:**
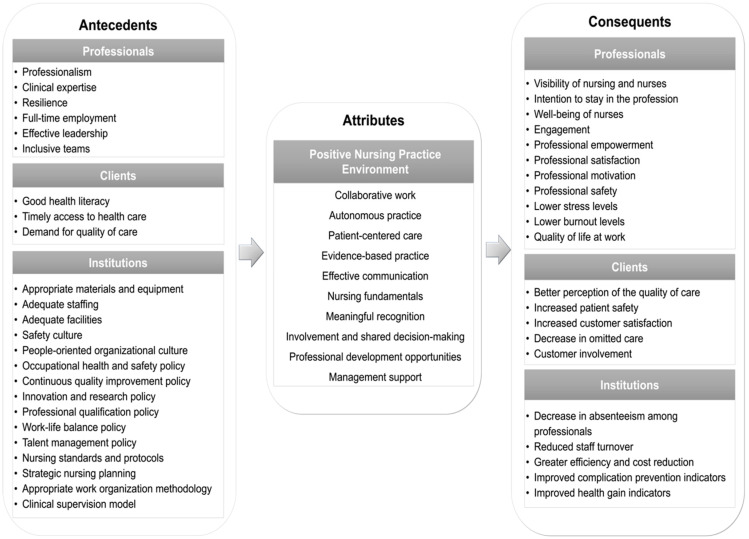
Model of the ‘Positive Nursing Practice Environment’ concept according to the methodology proposed by Walker and Avant.

According to Walker and Avant [20], the defining attributes of a concept are its essential characteristics. Our analysis identified 10 key attributes for the concept of ‘Positive Nursing Practice Environment’. These attributes are illustrated in Figure 2, which presents the model of the concept based on Walker and Avant’s methodology.

#### 3.2.1. Collaborative Work

Effective collaboration between nurses and other healthcare professionals is a cornerstone of a ‘Positive Nursing Practice Environment’. Such an environment, in turn, fosters collaborative work [23,24,25,26]. This collaboration is characterized by open communication, teamwork, mutual respect, shared responsibility, trust, recognition of roles, shared resources, and support, allowing each professional to be heard and respected [23,27,28,29]. Collegial relationships and collaboration are also linked to lower levels of burnout and fewer medication errors [24,30]. For instance, Poghosyan et al. [31] found that a positive work environment and strong collegial relationships improved chronic illness management, reducing emergency department visits and hospitalizations.

#### 3.2.2. Autonomous Practice

Autonomy is crucial in promoting a ‘Positive Nursing Practice Environment’ as it enables timely decision-making and enhances care quality [25,29,32,33]. Nurse autonomy is closely tied to teamwork, organizational support, and collaborative relationships [18]. A lack of autonomy can negatively impact job satisfaction, quality of care, and emotional well-being, contributing to burnout [34,35,36]. To support autonomous practice, institutions must provide opportunities for professional development and growth [37].

#### 3.2.3. Patient-Centered Care

Patient-centered care requires addressing individual patient needs effectively. The quality of care and nurse-patient interactions are significantly influenced by the nursing practice environment, particularly in terms of teamwork and safety [38,39]. Institutions that prioritize patient- and family-centered care have shown improvements in care quality and patient empowerment, contributing to a ‘Positive Nursing Practice Environment’ [32,39].

#### 3.2.4. Evidence-Based Practice

Access to information, resources, and support is vital for fostering evidence-based practice, which enhances self-efficacy, job satisfaction, and professional success [40]. A ‘Positive Nursing Practice Environment’ encourages the use of the latest scientific evidence to inform decisions, promoting teamwork, care quality, and the retention of qualified professionals [40]. Nursing leaders play a critical role in facilitating the adoption of evidence-based practices [23,29,32].

#### 3.2.5. Effective Communication

Effective communication is a fundamental component of a ‘Positive Nursing Practice Environment’ and is critical for delivering high-quality care [14,41]. Our research highlights the strong connection between communication and a positive environment, emphasizing the reciprocal impact they have on each other [14,27,28,42]. Open and respectful communication among healthcare professionals is essential for collaboration and care quality [29,31].

#### 3.2.6. Nursing Fundamentals

The fundamentals of nursing are essential to creating a ‘Positive Nursing Practice Environment’ [3,43]. A strong foundation in nursing philosophy and care models enhances care quality and aligns personal and organizational values, leading to greater professional satisfaction [25,43].

#### 3.2.7. Meaningful Recognition

Recognizing and valuing nurses for their contributions is crucial for fostering a ‘Positive Nursing Practice Environment’ [44]. Nurse managers play a key role in acknowledging nurses’ efforts, whether through formal recognition programs, flexible working hours, or professional development opportunities [23,24,25,44,45,46].

#### 3.2.8. Involvement and Shared Decision-Making

A ‘Positive Nursing Practice Environment’ requires shared governance, which empowers nurses to participate in institutional management and decision-making [34,43,47,48]. Involvement in governance has been linked to increased job satisfaction, autonomy, and reduced burnout [27,34,43].

#### 3.2.9. Professional Development Opportunities

Promoting personal and professional growth is fundamental for the success of both nurses and institutions [49,50]. Institutions can foster a ‘Positive Nursing Practice Environment’ by offering clinical orientation, regular training, and educational opportunities [3,49,50].

#### 3.2.10. Management Support

Management support is vital for creating and sustaining a ‘Positive Nursing Practice Environment’ [23,28,41,51,52]. Managers can support nurses by fostering collaboration, promoting evidence-based practices, and creating a culture of safety [23,41,51]. Positive management support enhances productivity, job satisfaction, and retention [41].

### 3.3. Model Case

According to Walker and Avant [20], a model case serves as a useful tool for illustrating the essential characteristics of a concept and may be derived from real-life situations, literary sources, or created by the researcher. In this study, the researchers developed a model case by synthesizing insights from existing literature and real-world institutions, ensuring that all key attributes of the concept were incorporated.

In the context of Clinical Practice X, there has been a persistent concern among managers to promote a favorable, safe, and healthy environment, which includes preventing physical injuries and creating a psychologically safe environment [53]. Growing evidence indicates the positive repercussions of this investment on clients, nurses, and the institution itself.

Patient care focuses on meeting individual needs. Collaborative work and effective communication ensure continuity of care; in this context, “everyone feels that they have a voice and can express their ideas” [53]. Investment in autonomous practices, evidence-based practice, and fundamental nursing principles contributes to the quality and safety of care. Valuing professionals and management support has culminated in active participation and shared decision-making, “having the right person in the right position, with the appropriate qualifications, experience, education, and training” [53]. Recognition and opportunities for professional development have motivated professionals to contribute more and remain with the institution and the profession. 

### 3.4. Additional Cases

The sixth stage of the Walker and Avant concept analysis involves defining additional cases to clarify the attributes that define the concept [20]. These cases include borderline, related, or contrary cases. To enhance clarity, this study presents both a related case and a contrary case.

#### 3.4.1. Related Case

While there are similarities between the concept being studied and the related case, and some important attributes are present, it does not meet all the defining characteristics outlined by Walker and Avant [20].

In Clinical Practice Z, the client is the focus. Care design and delivery are based on nursing fundamentals and best scientific evidence. Effective communication among professionals ensures continuity of care. Patient satisfaction with the care provided is high. To ensure quality and safety, nurses are encouraged to engage in autonomous practices, although collaboration among healthcare team members is not neglected. However, management support is inadequate, resulting in limited recognition and involvement of nurses in decision-making. Nurses feel that the leaders do not offer enough support or are not accessible, which leads to dissatisfaction [34]. Professional development opportunities for nurses are also minimal.

#### 3.4.2. Contrary Case

According to Walker and Avant [20], a contrary case represents the opposite of the concept’s meaning. This case lacks all defining attributes of the concept. Considering this aspect is crucial, as defining attributes are necessary to create a clear understanding of the concept and distinguish it from related concepts. Therefore, this case does not exemplify the concept under study.

In Clinical Context Y, resource management and cost control have been a primary concern for management boards. Care primarily focuses on the disease and results from the individual efforts of each professional group, with a lack of effective communication among professionals. Consequently, care is fragmented and often adheres to existing service routines without regard for evidence-based practice.

Due to the neglect of nursing fundamentals, there is a noticeable lack of investment in autonomous practices. Recognition and participation in decision-making are absent, and professional development opportunities are scarce. The lack of management support perpetuates many of the weaknesses in this professional practice environment. Nurses are not involved in decisions about hospital management, and there is a negative perception regarding the insufficiency of the team and available resources, which is associated with a perception of low support from management [34].

### 3.5. Antecedents

Antecedents are events or conditions that must occur before a particular concept can manifest and exist [20]. For the concept of ‘positive nursing practice environment’, antecedents can be categorized into three groups: those related to professionals, clients, and institutions.

#### 3.5.1. Professionals

Quality care delivery relies on highly qualified professionals who engage in continuous self-reflection and improvement [43,46]. Nurses with high levels of professionalism exhibit empowerment, motivation, commitment, and ethical behavior, positively influencing the nursing practice environment [51]. Research indicates that nurses with higher levels of education and knowledge are associated with better outcomes in terms of the working environment [14], reduced burnout and turnover due to increased responsibilities [33], and are more likely to report adverse events [54].

Clinically experienced nurses can integrate their knowledge, skills, attitudes, and values to enhance the workplace. Manning and Jones [51] found that clinical expertise strengthens competencies and knowledge, contributing to a ‘positive nursing practice environment’. Additionally, nurses with extensive clinical experience possess better knowledge of internal communication systems and the institution, further contributing to a ‘positive nursing practice environment’ [55]. Resilience, defined as the capacity to endure and overcome challenges [56], is another professional characteristic that influences the nursing practice environment. Research indicates that resilience can improve nurse retention by mitigating the negative effects of the workplace on well-being [56]. Moreover, nurses’ psychosocial factors shape their perception of the work environment and impact the nursing practice environment [49].

Studies show that full-time nurses with stable employment relationships tend to view their work environment more positively [57]. In contrast, part-time nurses often report reduced access to work resources, autonomy, and professional growth opportunities [46,54].

Effective leadership is critical in shaping the nursing practice environment. It involves engaging professionals, defining nursing values and ethical practice, and building trust and unity within the multidisciplinary team through collaboration and teamwork [45,58]. Leaders must exhibit self-awareness, visibility, accessibility, clear and transparent communication, recognition of professionals, encouragement of involvement and personal growth [30,45,59], and empowerment for exemplary practice [44]. Leadership influences professional retention, colleague relationships, job satisfaction, workload, and ultimately, the creation of a ‘positive nursing practice environment’ [18,30,59].

Nurse managers should strive for impartiality and create a non-discriminatory environment with inclusive teams. Developing cultural competence is essential for understanding nurses’ care behaviors and practices and making informed decisions to promote quality patient care and foster positive relationships [60]. Accommodating religious and cultural practices for immigrant nurses significantly improves their well-being and job satisfaction, reflecting an organizational culture that values diversity and supports its workforce [60]. Additionally, awareness of intergenerational differences among nurses is crucial for effective management. Nursing teams often consist of multiple generations, each with unique characteristics. Enhancing job satisfaction among nurses of varying nationalities, ages, and educational backgrounds should be part of the quality monitoring strategy [33].

#### 3.5.2. Clients

Increased life expectancy has led to a rise in the number and complexity of chronic diseases [24]. This shift has significant implications for health systems and policymakers, particularly regarding resource allocation and the development of specific therapeutic interventions. Access to healthcare and the provision of quality care are critical for improving chronic disease management and avoiding unnecessary healthcare utilization. However, various obstacles, including discriminatory practices and administrative, cultural, and linguistic barriers, hinder timely access to health services [24,60]. These obstacles contribute to an overloaded health system and a challenging work environment for nurses. Adequate information and knowledge about the health system enhance care quality. Patients who are well-informed and possess good health literacy are more likely to be engaged as partners in their care [60].

#### 3.5.3. Institutions

Appropriate material resources and equipment are essential for motivating professionals, ensuring their well-being, and delivering quality care [25,30,57]. Insufficient resources can lead to work task difficulties, professional burnout, and increased turnover [25,28]. Technology is a critical resource category that can enhance nurses’ ability to perform their duties. Notable examples include closed-circuit television cameras in critical patient services and advanced patient transfer equipment [50]. Information systems, electronic medical records, and patient education websites can also save significant time and effort for nurses [34]. During the COVID-19 pandemic, hospitals demonstrated greater investment in material resources and equipment due to increased needs [47]. Utilizing standardized languages, electronic communications, and training professionals to use available resources effectively are additional aspects contributing to a ‘positive nursing practice environment’ [34,46].

The quality of patient care is influenced by adequate facilities [57], including appropriate lighting, ventilation, temperature, cleanliness, and decoration [39,50,60]. However, long corridors and distances between patient rooms or nursing stations can impede nursing efficiency [38]. Architectural and ergonomic standards should be considered when designing medication preparation and administration spaces, ensuring safe care for both patients and healthcare professionals [50].

Staffing levels relative to patient numbers and their complexity significantly affect care quality and safety, as supported by numerous studies [33,58]. Adequate staffing reduces working hours and leave, improves inter-professional collaboration [50], and enhances personal and professional satisfaction, efficacy, and performance [3,43], leading to better patient and nurse outcomes [58,61].

A safety culture that prioritizes communication, trust, and transparency between healthcare professionals and patients is essential for fostering a ‘positive nursing practice environment’ [41,50]. Alongside safety culture, a positive safety climate and safety policies are established through organizational values that prioritize patient and professional safety, effective planning, and policy development to minimize conflict-induced errors [18,59].

Implementing occupational health policies and promoting a calm, non-stressful workplace with professional support and a pleasant atmosphere are crucial [35,49,50]. Addressing occupational stress factors and nurses’ psycho-emotional responses can improve occupational safety. Additionally, fostering a culture of trust, support, and fairness while eliminating discrimination can lead to more satisfied and committed nursing staff [50].

An organizational culture that emphasizes support and is people-oriented is vital. The power of organizational culture is substantial, as it can promote favorable working conditions and encourage healthcare professionals to deliver high-quality care [31,46]. Investing in organizational support through effective communication and involving nurses in decision-making significantly influences how nurses perceive their work environment, leading to increased satisfaction, motivation, and performance [18,31].

Organizational cultures that encourage innovative thinking and research are essential in cultivating a ‘positive nursing practice environment’, motivating nurses to provide high-quality care [31,62]. Healthcare institutions affiliated with academic entities often have more competent, knowledgeable, and skilled nurses engaged in research and teaching [28,50,54]. Implementing quality improvement programs and nursing care quality standards fosters organizational characteristics.

Talent management policies are a significant factor that affects the nursing work environment. Such policies promote the growth, development, and recognition of professionals [27,48]. When nurses work in an enabling environment that provides institutional support and learning opportunities, they are empowered to assume greater responsibility and promote professional autonomy [27,48].

The presence of a clinical supervision model in an organization leads to higher quality care and greater job satisfaction among healthcare professionals [33,48]. To achieve a ‘positive nursing practice environment’, it is essential to have mentoring and preceptorship programs in place. These programs also play a crucial role in shaping the nursing profession and developing evidence-based practice policies over time [27,35]. Clinical mentoring programs are particularly important for newly hired or intern nurses, as they provide an opportunity for them to interact with experienced nurses who ideally share the same culture or nationality [33,57]. It has been observed that active nurse development, mentoring, and training programs have been observed to be positively associated with higher levels of job performance, motivation, and job satisfaction [3].

Professional qualification policies can help nurses acquire and improve their skills in care planning and execution, decision-making, and creativity [3]. Implementing such qualification and self-improvement programs can provide an opportunity for nurses to hone their competencies and expertise, leading to better patient outcomes and organizational success.

In healthcare institutions, nursing leaders who create a ‘positive nursing practice environment’ involve nurses in setting goals and contributing to planning for nursing strategy planning [3,38]. Nurses who are dedicated to their work and show concern attach importance to planning and strive to improve their work performance [38]. Strategic nursing planning, which involves nurses, helps reduce conflicts and errors, leading to safer care, satisfied clients, and better patient outcomes [27].

Having nursing standards and protocols with clear procedures and instructions, using standardized language, and developing guidelines can enhance the quality and safety of the care provided. This, in turn, creates a ‘positive nursing practice environment’ that increases job satisfaction, motivates professionals, and improves retention in both the institution and the profession [48,49].

### 3.6. Consequences

Consequences refer to the outcomes or events that arise as a result of a particular concept, either as direct effects or follow-up results [20]. Beyond the antecedents, a ‘Positive Nursing Practice Environment’ has various consequences impacting professionals, clients, and institutions.

#### 3.6.1. Professionals

Institutions with a positive nursing practice environment clearly define the role of nurses and enhance their visibility and the visibility of the nursing profession [27]. Strategies such as management support, nurse involvement in governance, promotion of autonomous practices, shared decision-making, and inclusion in important committees and working groups can help recognize and advance the roles and skills of nurses [61,63]. These strategies are crucial for motivating nurses to remain in their positions and in the nursing profession [30,58]. 

A stimulating work environment with management support, adequate material and technological resources, appropriate workloads, and recognition from peers and the multidisciplinary team fosters nurse empowerment, high satisfaction levels, motivation, and a reduced intention to leave their jobs [29,49,55]. Professional development opportunities and a supportive organizational climate are also critical for encouraging nurses to stay and are strongly related to professional empowerment [59,63,64].

Training and empowering professionals can lead to increased productivity, commitment, and engagement [29,36,50,51]. Nurse managers play a pivotal role in nurturing a positive nursing practice environment [29,33,50,52]. Involving nurses in institutional and service management decisions enhances their commitment, productivity, and satisfaction [33,37,49]. Job satisfaction is associated with improved performance, fostering collaborative relationships, greater self-efficacy, autonomy, and higher levels of personal achievement and organizational commitment [30,33,49].

A positive nursing practice environment promotes professional motivation, influenced by factors such as resource accessibility, workload, professional development opportunities, autonomy, and fair evaluation systems [33,49]. It also ensures professional safety by reducing occurrences of verbal and physical abuse, discrimination, sexual harassment, and injuries such as musculoskeletal injuries, accidental bites, exposure to blood or body fluids, anxiety, and sleep disorders [18,59,65]. Enhanced professional safety reduces stress levels and burnout, thereby improving overall well-being [49,57,65].

The nursing practice environment can be a significant source of stress, impacting job satisfaction and well-being [47,66]. When nurses perceive a positive work environment, their quality of work life improves, with lower stress, anxiety, compassion fatigue, emotional exhaustion, and burnout [23,30,67]. The American Association of Critical Care Nurses (AACN) highlights the importance of healthy workplaces that balance professional and personal life while safeguarding the health and safety of professionals [49]. Thus, recognizing and fostering a positive nursing practice environment is fundamental.

#### 3.6.2. Clients

The nursing practice environment significantly affects clients, influencing their perception of care quality, which is closely related to timely, effective, efficient, and equitable care [31,36,68]. A positive nursing practice environment has been linked to higher client satisfaction and improved perceptions of care quality and safety [31,68,69]. Factors such as safety culture and communication, crucial to patient safety, help reduce errors and adverse events [36,67]. Effective communication not only enhances safety-related indicators but also facilitates interactions with patients and their families, increasing their satisfaction with care [14].

Omitted care, often associated with the nursing practice environment, can be influenced by workloads, staff adequacy, professional satisfaction, and participation and typically involves care related to mobilization, oral hygiene, and documentation [69].

#### 3.6.3. Institutions

Nursing practice environments impact various health indicators, including health gains and complication prevention [30,31,54]. A positive nursing practice environment correlates with improved pain control, reduced falls, and lower rates of healthcare-associated infections, sepsis, and pressure ulcers [23,31,36]. Our research indicates that such an environment also improves indicators such as 30-day mortality, intensive care unit admissions, length of stay, readmissions, adverse events, and medication errors, all related to patient safety [54,59]. These indicators affect institutional efficiency and can reduce care costs [31,49].

Absenteeism and turnover, which impact institutional productivity and efficiency, have clear financial repercussions [31,49]. A positive nursing practice environment can reduce sick leave and turnover intention. Nurses who are satisfied with their work environment and well-being are less likely to fall ill, resulting in decreased absenteeism and turnover [33,59]. Costs associated with turnover include pre-hire expenses (advertising, recruitment, interviewing, hiring, and agency nurses) and post-hire costs (onboarding, orientation, training, and decreased productivity) [33,44].

### 3.7. Empirical References

The final step of the analysis involves defining the empirical referents of the concept. Empirical referents help recognize or measure the defining characteristics or attributes of the concept. These referents are directly related to the defining attributes and are crucial for practice as they provide clear, observable phenomena to determine the concept’s existence [20].

Our research identified several tools for evaluating nursing practice environments and their dimensions. The Practice Environment Scale of the Nursing Work Index (PES-NWI) and the Nursing Work Index-Revised (NWI-R) are the most widely used instruments. The PES-NWI assesses work environment factors that support or hinder nurses’ ability to provide quality care, using a 1 to 4 Likert scale. This 31-item tool identifies a professional practice environment through five key dimensions: nurses’ participation in hospital affairs, basis for quality care, nurse manager capacity and leadership support, adequacy of staff and resources, and nurse-doctor relationship [12,68]. The NWI-R examines organizational characteristics of institutions using a 4-point Likert scale and includes 57 items across four subscales: autonomy, control over the practice context, nurse-doctor relationship, and organizational support for nurses [34,62].

### 3.8. Definition of the Concept

Following a comprehensive literature analysis, we have formulated a clear and concise definition of the concept, focusing on its applicability in nursing. The definition is intended to serve as a reference for healthcare professionals engaged with this concept.

A ‘positive nursing practice environment’ is defined as a work environment that is conducive to high-quality care, supportive of professionals, involves them in decision-making, fosters collaboration and effective communication, promotes autonomy and professional development, and is based on the fundamental principles of nursing and the best available evidence. It is also safe for both professionals and patients and prioritizes person-centered care.

## 4. Discussion

In recent decades, nursing practice environments have garnered significant interest [5,58,68]. Organizations have increasingly focused on improving working conditions and environments for nurses. In 2007, the International Council of Nurses (ICN) underscored the need to emphasize this area and outlined several essential characteristics of a positive work environment [16]. These characteristics include innovation policies for recruitment and retention, strategies for continuous education and professional development, adequate compensation, recognition programs, sufficient equipment and resources, and a safe care environment. Our study has identified these characteristics and emphasized professional development opportunities and recognition as key attributes of a ‘positive nursing practice environment’.

The Nursing Organizations Alliance has delineated several elements crucial to a healthy working environment [70]. These elements include a culture of collaborative practice, effective communication, accountability with clearly defined roles, an adequate number of qualified professionals, competent leadership, shared decision-making, and continuous professional development. These elements align with the attributes and antecedents identified in our concept analysis of a ‘positive nursing practice environment’, reinforcing our findings.

The Registered Nurses’ Association of Ontario (RNAO) has developed the Healthy Work Environments Best Practice Guidelines and introduced the Patient Care Delivery Systems Model to enhance staffing and workload practices [17]. This model incorporates inputs related to patients/clients, nurses, and system characteristics, along with their interactions. These inputs, together with critical nursing processes such as models of care, nursing leadership, infrastructures, and environmental complexity, result in various outcomes for patients, providers, and systems.

Our concept analysis reveals that the inputs related to clients, nurses, and system characteristics are interconnected. Some inputs identified in our study correspond with those outlined by the RNAO, such as perceptions of care quality, health knowledge, and entry points for admissions. The RNAO also recognizes nurse characteristics such as professional status, employment status, education, experience, and competence [17]. Additionally, system characteristics and behaviors identified by RNAO align with our findings, including staffing ratios, available resources, and shared decision-making. RNAO identifies throughputs like care models, leadership styles, and team characteristics [17]. Outputs related to a ‘positive nursing practice environment’ encompass client, nurse, and system outcomes, including readmission rates, patient satisfaction, job satisfaction, professional and client safety, nurse retention, cost, quality of care, absenteeism, and error rates.

The Magnet Hospital Recognition Program for Nursing Service Excellence, established by the American Nurses Association, identified key attributes of healthcare institutions excelling in nurse recruitment and retention, categorized into five components: transformational leadership, structural empowerment, exemplary professional practice, new knowledge and innovations, and empirical results [9,71]. The attributes considered in these components include leadership quality, organizational structure, management style, personnel policies, care models, quality of care, improvement processes, resources, autonomy, nursing image, interdisciplinary relationships, and professional development [9,13,71].

Our concept analysis of a ‘positive nursing practice environment’ aligns with several characteristics identified by the Magnet program, including leadership, professional development policies, organizational culture, human resources policies, nursing fundamentals, clinical supervision models, quality and safety concerns, autonomous practices, and collaborative work practices.

Recently, the World Health Organization (WHO) published a report analyzing the activities and outcomes of the International Year of the Nurse and the Midwife (2019–2021) [72]. This report highlights various initiatives undertaken by countries to enhance nurses’ working environments, underscoring the relevance and importance of this work.

The alignment between the characteristics identified in the concept of a ‘positive nursing practice environment’ and those in various reference documents demonstrates that the nursing work environment is a dynamic and multifaceted space, shaped by a diverse array of defining characteristics. This concept analysis facilitates a clear systematization of these characteristics, which significantly impact professionals, patients, and institutions.

A notable limitation of this study is the concentration of research conducted primarily within hospital settings. Although we incorporated studies from specialized areas such as primary health care, school health, and military nursing, there may be relevant insights from other contexts that were not covered. The cultural perspective of this concept may also be considered a limitation of this study. However, efforts were made to minimize this limitation by conducting an extensive search without language restrictions and including studies from various countries, thereby capturing diverse cultural aspects. Despite this, the identification of the key characteristics of a ‘positive nursing practice environment’ enables the development of a framework that can drive continuous improvements in nursing environments and enhance the quality and safety of patient care.

## 5. Conclusions

The conceptual analysis of a ‘positive nursing practice environment’ offers a substantial contribution to understanding and defining the conditions that support optimal nursing practice. The analysis involved a comprehensive review of the extensive literature, which presented challenges in terms of data extraction and categorization. Nonetheless, including all studies that met the inclusion criteria provided a broad perspective on the topic, revealing a wide array of characteristics and outcomes associated with a ‘positive nursing practice environment’.

This analysis provides a clear and defined framework for understanding the concept, which will support future research and initiatives aimed at optimizing nursing practice environments. By elucidating and specifying the attributes of a ‘positive nursing practice environment’, this study establishes a foundation for ongoing advancement and improvement in the field.

## Figures and Tables

**Table 1:** Database search strategy and results.

Database (Host)	Search String	Results
Medline (PubMed)	((“workplace” [MeSH Terms] OR “work environment” [Title/Abstract] OR “work setting” [Title/Abstract]) AND (“healthy” [Title/Abstract] OR “favorable” [Title/Abstract] OR “positive work environment” [Title/Abstract]) AND (“nursing” [MeSH Terms] OR “nurs*” [Title/Abstract] OR “nursing staff” [MeSH Terms] OR “nurses” [MeSH Terms] OR “nursing practice” [Title/Abstract]))	636
CINAHL Complete (EBSCO)	((AB (nurs* OR nursing practice) OR (MH “nursing care”) OR (MH “staff nurses”) OR (MM “nurses”)) AND (AB (work environment OR work setting OR workplace) OR (MM “work environment”) OR (MH “professional practice”)) AND (AB (healthy OR favorable OR positive work environment))	997
Mediclatina (EBSCO)	((AB (nurs* OR nursing practice) OR (MH “nursing care”) OR (MH “staff nurses”) OR (MM “nurses”)) AND (AB (work environment OR work setting OR workplace) OR (MM “work environment”) OR (MH “professional practice”)) AND (AB (healthy OR favorable OR positive work environment))	12
RCAAP—Portugal’s Open Access Scientific Repository	(nurs* [description] AND work environment AND (healthy OR favorable OR positive work environment)) Filter: Dissertations and theses	232
WorldCat	(kw: “nurs*” AND kw: “work environment” AND kw: (“healthy” OR “favorable” OR “positive”)) Filter: Dissertations and theses	264

* Truncation.

## Data Availability

Dataset available on request from the authors.

## Supplementary Materials

The following supporting information can be downloaded at: https://www.mdpi.com/article/10.3390/nursrep14040222/s1, Table S1: Included studies.

## Public Involvement Statement

No public involvement in any aspect of this research.

## Guidelines and Standards Statement

This manuscript was drafted against the PRISMA-ScR.
