# Supplementary material for: Positive Nursing Practice Environment: A Concept Analysis
Source: Nurs Rep. 2024 Oct 17;14(4):3052–68. doi: 10.3390/nursrep14040222 (PMC11503395; doi:10.3390/nursrep14040222)
Supplement: Supplementary file 1 [file nursrep-14-00222-s001.zip › nursrep-3211282-supplementary.pdf]

**Supplementary Materials**

## Table S1 – Included Studies

| |
|---|
| Cortelyou-Ward, K. H. (2007). *Work environment and the effect on occupational commitment and intent to leave: a study of bedside registered nurses*. https://www.worldcat.org/pt/title/950400102 |
| Parsons, M. L., Clark, P., Marshall, M., & Cornett, P. A. (2007). Team behavioral norms: a shared vision for a healthy patient care workplace. *Critical care nursing quarterly*, *30*(3), 213–218. https://doi.org/10.1097/01.CNQ.0000278921.14197.f9 |
| Manojlovich, M., DeCicco, D. (2007). Healthy Work Environments, Nurse-Physician Communication, and Patients' Outcomes. *Am J Crit Care*, 16 (6): 536–543. https://doi.org/10.4037/ajcc2007.16.6.536 |
| Lavoie-Tremblay, M., Wright, D., Desforges, N., Gélinas, C., Marchionni, C., & Drevniok, U. (2008). Creating a healthy workplace for new-generation nurses. *Journal of Nursing Scholarship : An Official Publication of Sigma Theta Tau International Honor Society of Nursing*, *40*(3), 290–297. https://doi.org/10.1111/j.1547-5069.2008.00240.x |
| Al-Ateeq, E. (2008). The relationship between registered nurses' perceptions of their work environment and their perceptions of patient safety culture (Order No. 3321161). (304341024). Retrieved from https://www.proquest.com/dissertations-theses/relationship-between-registered-nurses/docview/304341024/se-2 |
| Kotzer, A. M., & Arellana, K. (2008). Defining an evidence-based work environment for nursing in the USA. *Journal of Clinical Nursing*, *17*(12), 1652–1659. https://doi.org/10.1111/J.1365-2702.2007.02148.X |
| Beal, J. A., Riley, J. M., & Lancaster, D. R. (2008). Essential elements of an optimal clinical practice environment. *The Journal of Nursing Administration*, *38*(11), 488–493. https://doi.org/10.1097/01.NNA.0000339475.65466.d2 |
| M, Meraviglia, M., Grobe, S. J., Tabone, S., Wainwright, M., Shelton, S., Miner, H., & Jordan, C. (2009). Creating a positive work environment: implementation of the nurse-friendly hospital criteria. *The Journal of Nursing Administration*, *39*(2), 64–70. https://doi.org/10.1097/NNA.0b013e318195a82b |
| Stichler, J.F. (2009). Healthy, healthful, and healing environments: a nursing imperative. *Critical Care Nursing Quarterly*, *32*(3), 176–188. https://doi.org/10.1097/CNQ.0b013e3181ab9149 |
| Helton, R.E. (2009). Creating a healthy work environment. Critical Care Nurse, 29(5), 78–79, 80. https://doi.org/10.4037/ccn2009716 |

| |
|---|
| Cohen, J., Stuenkel, D. & Nguyen, Q. (2009). Providing a healthy work environment for nurses: the influence on retention. Journal of Nursing Care Quality, 24(4), 308–315. https://doi.org/10.1097/NCQ.0b013e3181a4699a |
| Shirey M. R. (2009). Authentic leadership, organizational culture, and healthy work environments. Critical care nursing quarterly, 32(3), 189–198. https://doi.org/10.1097/CNQ.0b013e3181ab91db |
| Kramer, M., Schmalenberg, C., & Maguire, P. (2010). Nine structures and leadership practices essential for a magnetic (healthy) work environment. Nursing administration quarterly, 34(1), 4–17. https://doi.org/10.1097/NAQ.0b013e3181c95ef4 |
| Weston, Marla J,PhD., R.N. (2010). Strategies for enhancing autonomy and control over nursing practice. Online Journal of Issues in Nursing, 15(1), 12-1G,2G,3G,4G,5G,6G,7G,8G,9G,10G,11G,12G. Retrieved from https://www.proquest.com/scholarly-journals/strategies-enhancing-autonomy-control-over/docview/722130651/se-2 |
| Sherman, R. & Pross, E., (2010). Growing Future Nurse Leaders to Build and Sustain Healthy Work Environments at the Unit Level. OJIN: The Online Journal of Issues in Nursing Vol. 15, No. 1, Manuscript 1. https://doi.org/10.3912/OJIN.Vol15No01Man01 |
| Ostermann, T., Bertram, M., & Büssing, A. (2010). A pilot study on the effects of a team building process on the perception of work environment in an integrative hospital for neurological rehabilitation. BMC complementary and alternative medicine, 10, 10. https://doi.org/10.1186/1472-6882-10-10 |
| Kupperschmidt, B., Kientz, E., Ward & J., Reinholz, B. (2010). A Healthy Work Environment: It Begins With You. *OJIN: The Online Journal of Issues in Nursing* Vol. 15, No. 1, Manuscript 3. https://doi.org/10.3912/OJIN.Vol15No01Man03 |
| Patrician, P. A., Shang, J., & Lake, E. T. (2010). Organizational determinants of work outcomes and quality care ratings among Army Medical Department registered nurses. Research in nursing & health, 33(2), 99–110. https://doi.org/10.1002/nur.20370 |
| Zangaro, G. A., & Kelley, P. A. (2010). Job satisfaction and retention of military nurses: a review of the literature. Annual review of nursing research, 28, 19–41. https://doi.org/10.1891/0739-6686.28.19 |
| Nantsupawat, A., Srisuphan, W., Kunaviktikul, W., Wichaikhum, O. A., Aungsuroch, Y., & Aiken, L. H. (2011). Impact of nurse work environment and staffing on hospital nurse and quality of care in Thailand. Journal of nursing scholarship : an official publication of Sigma Theta Tau International Honor Society of Nursing, 43(4), 426–432. https://doi.org/10.1111/j.1547-5069.2011.01419.x |
| Purdy, N., Spence Laschinger, H. K., Finegan, J., Kerr, M., & Olivera, F. (2010). Effects of work environments on nurse and patient outcomes. Journal of nursing management, 18(8), 901–913. https://doi.org/10.1111/j.1365-2834.2010.01172.x |

Kramer, M., Maguire, P., & Brewer, B. B. (2011). Clinical nurses in Magnet hospitals confirm productive, healthy unit work environments. Journal of nursing management, 19(1), 5–17. https://doi.org/10.1111/j.1365-2834.2010.01211.x

Ritter D. (2011). The relationship between healthy work environments and retention of nurses in a hospital setting. Journal of nursing management, 19(1), 27–32. https://doi.org/10.1111/j.1365-2834.2010.01183.x

Anderson, K. A. (2011). The impact that leadership practices of the nurse manager and nursing practice environments have on job satisfaction of registered nurses in two urban teaching hospitals (Order No. 3576886). Available from Publicly Available Content Database. (1465367034). Retrieved from https://www.proquest.com/dissertations-theses/impact-that-leadership-practices-nurse-manager/docview/1465367034/se-2

Weber B. B. (2011). The role of professional nursing organizations in maintaining a healthy workplace. Plastic surgical nursing: official journal of the American Society of Plastic and Reconstructive Surgical Nurses, 31(3), 92–94. https://doi.org/10.1097/PSN.0b013e318227253c

Thomas, A. P. (2012). Relationship of a Healthy Work Environment to Retention of Direct Care Nurses in a Hospital Setting. Kennesaw State Universit.

England J. B. (2012). An integrative literature review : the relationship between healthy work environment and nursing-sensitive patient outcomes (dissertation).

Batson V. D. (2012). Managerial coaching and staff nurse perceptions of work environment professional work satisfaction job satisfaction and intent to stay (dissertation). University of Texas.

Pryse Y. M. & IUPUI ScholarWorks. (2012). Using evidence based practice : the relationship between work environment nursing leadership and nurses at the bedside (dissertation).

Blake, N., Mills, M., & Guerrero, V. (2012). Radiology nursing staff use the HWE assessment tool to improve the work environment. AACN advanced critical care, 23(2), 128–132. https://doi.org/10.1097/NCI.0b013e3182501cb8

Blake, N., Mills, M., & Guerrero, V. (2012). Radiology nursing staff use the HWE assessment tool to improve the work environment. AACN advanced critical care, 23(2), 128–132. https://doi.org/10.1097/NCI.0b013e3182501cb8

Averlid, G., & Axelsson, S. B. (2012). Health-promoting collaboration in anesthesia nursing: a qualitative study of nurse anesthetists in Norway. AANA journal, 80(4 Suppl), S74–S80.

Brady Schwartz, D., & Burnes Bolton, L. (2012). Leadership imperative: creating and sustaining healthy workplace environments. The Journal of nursing administration, 42(11), 499–501. https://doi.org/10.1097/NNA.0b013e3182714521

Boev C. (2012). The relationship between nurses' perception of work environment and patient satisfaction in adult critical care. Journal of nursing scholarship : an official publication of Sigma Theta Tau International Honor Society of Nursing, 44(4), 368–375. https://doi.org/10.1111/j.1547-5069.2012.01466.x

Kirwan, M., Matthews, A., & Scott, P. A. (2013). The impact of the work environment of nurses on patient safety outcomes: a multi-level modelling approach. International journal of nursing studies, 50(2), 253–263. https://doi.org/10.1016/j.ijnurstu.2012.08.020

Quinn, L. (2013). Throughput and nurses' workloads: Influences on nurse and patient outcomes (dissertation). University of Pennsylvania

Nayback-Beebe, A. M., Forsythe, T., Funari, T., Mayfield, M., Thoms, W., Jr, Smith, K. K., Bradstreet, H., & Scott, P. (2013). Using evidence-based leadership initiatives to create a healthy nursing work environment. Dimensions of critical care nursing : DCCN, 32(4), 166–173. https://doi.org/10.1097/DCC.0b013e3182998121

Dingley, J., & Yoder, L. (2013). The public health nursing work environment: review of the research literature. Journal of public health management and practice : JPHMP, 19(4), 308–321. https://doi.org/10.1097/PHH.0b013e31825ceadc

Ayamolowo et al. (2013). Job Satisfaction and Work Environment of Primary Health Care Nurses in Ekiti State, Nigeria: an Exploratory Study. International Journal of Caring Sciences. 6 (3). pp. 531-542.

Budin, W. C., Brewer, C. S., Chao, Y. Y., & Kovner, C. (2013). Verbal abuse from nurse colleagues and work environment of early career registered nurses. Journal of nursing scholarship : an official publication of Sigma Theta Tau International Honor Society of Nursing, 45(3), 308–316. https://doi.org/10.1111/jnu.12033

Blake, N., Leach, L. S., Robbins, W., Pike, N., & Needleman, J. (2013). Healthy work environments and staff nurse retention: the relationship between communication, collaboration, and leadership in the pediatric intensive care unit. Nursing administration quarterly, 37(4), 356–370. https://doi.org/10.1097/NAQ.0b013e3182a2fa47

Sanders, C. L., Krugman, M., & Schloffman, D. H. (2013). Leading change to create a healthy and satisfying work environment. Nursing administration quarterly, 37(4), 346–355. https://doi.org/10.1097/NAQ.0b013e3182a2fa2d

Kelly, D., Kutney-Lee, A., Lake, E. T., & Aiken, L. H. (2013). The critical care work environment and nurse-reported health care-associated infections. American journal of critical care : an official publication, American Association of Critical-Care Nurses, 22(6), 482–488. https://doi.org/10.4037/ajcc2013298

Kirwan, M., Matthews, A., & Scott, P. A. (2013). The impact of the work environment of nurses on patient safety outcomes: a multi-level modelling approach. International journal of nursing studies, 50(2), 253–263. https://doi.org/10.1016/j.ijnurstu.2012.08.020

Willingham A. R. (20141201080000). Meaningful recognition in a healthy work environment for nurse engagement in a critical care setting (dissertation). Kennesaw State University.

Rollins, L. (2014). Healthy Work Environment Orientation Training and Psychiatric Nurse Retention. (dissertations). Walden University

Sili, A., Fida, R., Trezza, T., Vellone, E., & Alvaro, R. (2014). Nurse coordinator leadership and work environment conflicts: consequences for physical and work-related health of nursing staff. La Medicina del lavoro, 105(4), 296–306.

Djukic, M., Kovner, C. T., Brewer, C. S., Fatehi, F., & Greene, W. H. (2014). Exploring direct and indirect influences of physical work environment on job satisfaction for early-career registered nurses employed in hospitals. Research in nursing & health, 37(4), 312–325. https://doi.org/10.1002/nur.21606

Choi, J., & Boyle, D. K. (2014). Differences in nursing practice environment among US acute care unit types: a descriptive study. International journal of nursing studies, 51(11), 1441–1449. https://doi.org/10.1016/j.ijnurstu.2014.03.001

Stalpers, D., de Brouwer, B. J., Kaljouw, M. J., & Schuurmans, M. J. (2015). Associations between characteristics of the nurse work environment and five nurse-sensitive patient outcomes in hospitals: a systematic review of literature. International journal of nursing studies, 52(4), 817–835. https://doi.org/10.1016/j.ijnurstu.2015.01.005

Finlay, C. M., Walsh, K. P., & Mills, K. H. (2014). Induction of regulatory cells by helminth parasites: exploitation for the treatment of inflammatory diseases. Immunological reviews, 259(1), 206–230. https://doi.org/10.1111/imr.12164

Bai J., Zhang Q., Wang Y., Yu L.-P., Pei X.-B., Cheng L. & Hsu L. (2015). Work environment for Chinese nurses in different types of ICUs: a multisite cross-sectional survey. Journal of Nursing Management 23, 498–509. https://doi.org/10.1111/jonm.12163

Blosky, M. A., & Spegman, A. (2015). Let's talk about it: Communication and a healthy work environment. Nursing management, 46(6), 32–38. https://doi.org/10.1097/01.NUMA.0000465398.67041.58

Nowrouzi, B., Lightfoot, N., Larivière, M., Carter, L., Rukholm, E., Schinke, R., & Belanger-Gardner, D. (2015). Occupational Stress Management and Burnout Interventions in Nursing and Their Implications for Healthy Work Environments: A Literature Review. Workplace health & safety, 63(7), 308–315. https://doi.org/10.1177/2165079915576931

Hildingsson, I., & Fenwick, J. (2015). Swedish midwives' perception of their practice environment - A cross sectional study. Sexual & reproductive healthcare : official journal of the Swedish Association of Midwives, 6(3), 174–181. https://doi.org/10.1016/j.srhc.2015.02.001

Skarbek, A. J., Johnson, S., & Dawson, C. M. (2015). A Phenomenological Study of Nurse Manager Interventions Related to Workplace Bullying. The Journal of nursing administration, 45(10), 492–497. https://doi.org/10.1097/NNA.0000000000000240

| |
|---|
| Bai J. (2016). Does job satisfaction mediate the relationship between healthy work environment and care quality?. Nursing in critical care, 21(1), 18–27. https://doi.org/10.1111/nicc.12122 |
| Tei-Tominaga, M., & Sato, F. (2016). Effect of nurses' work environment on patient satisfaction: A cross-sectional study of four hospitals in Japan. Japan journal of nursing science : JJNS, 13(1), 105–113. https://doi.org/10.1111/jjns.12091 |
| Sevilla-Zeigen, N. (2016). A grounded theory approach to healthy work environment : its impact on nurses patient safety and significance in healthcare setting (dissertation). University of San Diego. |
| Kilańska, D., Gorzkowicz, B., Sienkiewicz, Z., Lewandowska, M., Dominiak, I., & Bielecki, W. (2016). Evaluation of chosen determinants of the positive practice environments (PPE) at Polish nursing wards. Medycyna pracy, 67(1), 11–19. https://doi.org/10.13075/mp.5893.00225 |
| Cuff, L. (2016). Healthy Work Environment: Essentials for Outcome Improvement. (dissertation). Walden University |
| Friese, C. R., Siefert, M. L., Thomas-Frost, K., Walker, S., & Ponte, P. R. (2016). Using Data to Strengthen Ambulatory Oncology Nursing Practice. Cancer nursing, 39(1), 74–79. https://doi.org/10.1097/NCC.0000000000000240 |
| Augsburg College. (2016). Developing a healthy work environment for radiology nurses through metaphor (dissertation). Augsburg College |
| Blake, N., Oriza, N. S., Winter, V., & Imperial-Perez, F. (2016). Shared Governance for a Healthy Work Environment in a Pediatric Cardiothoracic Intensive Care Unit. AACN advanced critical care, 27(2), 152–157. https://doi.org/10.4037/aacnacc2016968 |
| Liu, J., You, L., Zheng, J., Ross, A. M., & Liu, K. (2016). Effects of Work Environment on Quality of Care in ICUs: A Multisite Survey in China. Journal of nursing care quality, 31(3), E1–E8. https://doi.org/10.1097/NCQ.0000000000000160 |
| Roque, S. (2016). Impacto do ambiente de prática de enfermagem na qualidade e segurança dos cuidados. (dissertation). Universidade Católica Portuguesa |
| Hinsley, K. E., Marshall, A. C., Hurtig, M. H., Thornton, J. M., O'Connell, C. A., Porter, C. L., Connor, J. A., & Hickey, P. A. (2016). Monitoring the health of the work environment with a daily assessment tool: the REAL - Relative Environment Assessment Lens - indicator. Cardiology in the young, 26(6), 1082–1089. https://doi.org/10.1017/S1047951115001808 |
| Huddleston, P., & Gray, J. (2016). Describing Nurse Leaders' and Direct Care Nurses' Perceptions of a Healthy Work Environment in Acute Care Settings, Part 2. The Journal of nursing administration, 46(9), 462–467. https://doi.org/10.1097/NNA.0000000000000376 |

| |
|---|
| Poghosyan, L., Liu, J., Shang, J., & D'Aunno, T. (2017). Practice environments and job satisfaction and turnover intentions of nurse practitioners: Implications for primary care workforce capacity. Health care management review, 42(2), 162–171. https://doi.org/10.1097/HMR.0000000000000094 |
| Numminen, O., Ruoppa, E., Leino-Kilpi, H., Isoaho, H., Hupli, M., & Meretoja, R. (2016). Practice environment and its association with professional competence and work-related factors: perception of newly graduated nurses. Journal of nursing management, 24(1), E1–E11. https://doi.org/10.1111/jonm.12280 |
| Kelly, L., & Todd, M. (2017). Compassion Fatigue and the Healthy Work Environment. AACN advanced critical care, 28(4), 351–358. https://doi.org/10.4037/aacnacc2017283 |
| Kral, A. (2017). The influence of work environment on workplace bullying and retention of new graduate nurses.. (dissertation). Bethel University |
| Al-Hamdan, Z., Manojlovich, M., & Tanima, B. (2017). Jordanian Nursing Work Environments, Intent to Stay, and Job Satisfaction. Journal of nursing scholarship : an official publication of Sigma Theta Tau International Honor Society of Nursing, 49(1), 103–110. https://doi.org/10.1111/jnu.12265 |
| Oliveira, E. M., Barbosa, R. L., Andolhe, R., Eiras, F. R., & Padilha, K. G. (2017). Nursing practice environment and work satisfaction in critical units. Ambiente das práticas de enfermagem e satisfação profissional em unidades críticas. Revista brasileira de enfermagem, 70(1), 79–86. https://doi.org/10.1590/0034-7167-2016-0211 |
| Swiger, P. (2017). The Military Nursing Practice Environment's Association with Patient Outcomes. (dissertations). |
| Santos, J. L. dos, Menegon, F. H. A., Pin, S. B. de, Erdmann, A. L., Oliveira, R. J. de, & Costa, I. A. P. (2017). The nurse’s work environment in a hospital emergency service. Rev Rene, 18(2), 195–203. https://doi.org/10.15253/2175-6783.2017000200008 |
| Jones, K. (2017). The benefits of Magnet status for nurses, patients and organisations. Nursing Times, 113(11). |
| Akter, N., Akkadechanunt, T., Chontawan, R., & Klunklin, A. (2018). Factors predicting quality of work life among nurses in tertiary-level hospitals, Bangladesh. International Nursing Review, 65(2), 182–189. https://doi.org/10.1111/INR.12401 |
| Kol, E., İlaslan, E., & Turkay, M. (2017). The effectiveness of strategies similar to the Magnet model to create positive work environments on nurse satisfaction. International Journal of Nursing Practice, 23(4). https://doi.org/10.1111/IJN.12557 |
| Fang, Y., & McDonald, T. (2018). Management capacity to promote nurse workplace health and safety. Journal of Nursing Management (John Wiley & Sons, Inc.), 26(3), 288–294. https://doi.org/10.1111/jonm.12544 |

| |
|---|
| Yoo, M. S., & Kim, K. J. (2017). Exploring the Influence of Nurse Work Environment and Patient Safety Culture on Attitudes Toward Incident Reporting. JONA: The Journal of Nursing Administration, 47(9), 434–440. https://doi.org/10.1097/NNA.0000000000000510 |
| Balsanelli, A. P., David, D. R., & Ferrari, T. G. (2018). Nursing leadership and its relationship with the hospital work environment. Acta Paulista de Enfermagem, 31(2), 187–193. https://doi.org/10.1590/1982-0194201800027 |
| Graystone, R. (2018). Creating the Framework for a Healthy Practice Environment. JONA: The Journal of Nursing Administration, 48(10), 469–470. https://doi.org/10.1097/NNA.0000000000000652 |
| Kim, K.-J., Yoo, M. S., & Seo, E. J. (2018). Exploring the Influence of Nursing Work Environment and Patient Safety Culture on Missed Nursing Care in Korea. Asian Nursing Research, 12(2), 121–126. https://doi.org/10.1016/j.anr.2018.04.003 |
| Wei, H., Sewell, K. A., Woody, G., & Rose, M. A. (2018). The state of the science of nurse work environments in the United States: A systematic review. International Journal of Nursing Sciences, 5(3), 287–300. https://doi.org/10.1016/J.IJNSS.2018.04.010 |
| Brofidi, K., Vlasiadis, K., & Philalithis, A. (2019). Greek hospital environments. International Journal of Health Care Quality Assurance (09526862), 32(3), 645–652. https://doi.org/10.1108/IJHCQA-05-2018-0102 |
| Cuduro, F. L. F., & de Macedo, S. M. K. (2018). Evaluation of the working environment between nursing professionals in an urgent and emergency unit. 17(2), 388–399. https://dx.doi.org/10.6018/eglobal.17.2.283991 |
| Harrison, G. D., & Zavotsky, K. E. (2018). Are critical care nurses more likely to leave after a merger? Nursing Management, 49(9), 32–39. https://doi.org/10.1097/01.NUMA.0000544462.60366.C3 |
| Hiler, C. A., Hickman, R. L., Jr, Reimer, A. P., & Wilson, K. (2018). Predictors of Moral Distress in a US Sample of Critical Care Nurses. American journal of critical care : an official publication, American Association of Critical-Care Nurses, 27(1), 59–66. https://doi.org/10.4037/ajcc2018968 |
| Silva, R. P. L. (2018). Ambiente de prática profissional, carga de trabalho e omissão de cuidados de enfermagem em Unidades de Terapia Intensiva. Dissertação de Mestrado, Escola de Enfermagem de Ribeirão Preto, Universidade de São Paulo, Ribeirão Preto. doi:10.11606/D.22.2019.tde-18032019-191531. |
| Liu, J., Zheng, J., Liu, K., & You, L. (2019). Relationship Between Work Environments, Nurse Outcomes, and Quality of Care in ICUs: Mediating Role of Nursing Care Left Undone. Journal of nursing care quality, 34(3), 250–255. https://doi.org/10.1097/NCQ.0000000000000374 |
| Nogueira, L. de S., Sousa, R. M. de, Guedes, E. de S., Turrini, R. N. T., Cruz, D. de A. L. M. da, & Santos, M. A. dos. (2018). Burnout and nursing work environment in public health institutions. Revista Brasileira de Enfermagem, 71(2), 336–342. https://doi.org/10.1590/0034-7167-2016-0524 |

Al-Hamdan, Z., Banerjee, T., & Manojlovich, M. (2018). Communication With Physicians as a Mediator in the Relationship Between the Nursing Work Environment and Select Nurse Outcomes in Jordan. Journal of nursing scholarship : an official publication of Sigma Theta Tau International Honor Society of Nursing, 50(6), 714–721. https://doi.org/10.1111/jnu.12417

Swiger, P. A., Loan, L. A., Raju, D., Breckenridge-Sproat, S. T., Miltner, R. S., & Patrician, P. A. (2018). Relationships between Army nursing practice environments and patient outcomes. Research in Nursing & Health, 41(2), 131–144. https://doi.org/10.1002/nur.21855

Santos, R. dos, Paiva, M. C. da S. de, & Spiri, W. C. (2018). Association between nurses' quality of life and work environment. Acta Paulista de Enfermagem, 31(5), 472–479. https://doi.org/10.1590/1982-0194201800067

Ulrich, B., Barden, C., Cassidy, L., & Varn-Davis, N. (2019). Critical Care Nurse Work Environments 2018: Findings and Implications. Critical Care Nurse, 39(2), 67–84. https://doi.org/10.4037/CCN2019605

Blake, N. (2019). Creating Healthy Work Environments: Our Voice, Our Strength. AACN Advanced Critical Care, 30(1), 65–67. https://doi.org/10.4037/aacnacc2019558

Halm, M. (2019). The Influence of Appropriate Staffing and Healthy Work Environments on Patient and Nurse Outcomes. American Journal of Critical Care : An Official Publication, American Association of Critical-Care Nurses, 28(2), 152–156. https://doi.org/10.4037/AJCC2019938

Albashayreh, A., Al Sabei, S. D., Al-Rawajfah, O. M., & Al-Awaisi, H. (2019). Healthy work environments are critical for nurse job satisfaction: implications for Oman. International nursing review, 66(3), 389–395. https://doi.org/10.1111/inr.12529

Chen, J., Ramalhal, T., & Bernardes Lucas, P. (2019). Ambiente de prática de enfermagem e a satisfação dos enfermeiros em contexto hospitalar: Pensar Enfermagem, 23(2), 29–42. https://doi.org/10.56732/PENSARENF.V23I2.161

Jones, L., Cline, G. J., Battick, K., Burger, K. J., & Amankwah, E. K. (2019). Communication Under Pressure: A Quasi-Experimental Study to Assess the Impact of a Structured Curriculum on Skilled Communication to Promote a Healthy Work Environment. Journal for nurses in professional development, 35(5), 248–254. https://doi.org/10.1097/NND.0000000000000573

Mrayyan, M. T. (2019). Nurses' views on hospital organizational characteristics. Nursing Forum, 54(4), 650–660. https://doi.org/10.1111/nuf.12390

Barandino, J.P., & Platon Soriano, G. (2019). Practice environment and work-related quality of life among nurses in a selected hospital in Zamboanga, Philippines: A correlational study. Nursing Practice Today, 6(4), 223–228. https://doi.org/10.18502/NPT.V6I4.1944

Moura, L. N. (2019). Ambiente de prática e empoderamento de enfermeiros de um hospital universitário. (dissertation). Universidade Federal de Santa Maria

Pinto, P. (2019). The influence of practice environments into Care individualization and omitting nursing care activities. (dissertation). University of Coimbra.

Ferreira, T. D. M. (2019). Ambiente da prática profissional da enfermagem entre instituições de saúde. Retrieved from: https://bdtd.ibict.br/vufind/Record/UNICAMP-30_346099a5f97d4452e3d52c644f764794

Cassidy, L. F. (2019). The Relationship among Healthy Work Environments, Nurse Caring, and Nursing-sensitive Patient Outcomes in Magnet Hospitals. Florida Atlantic University ProQuest Dissertations Publishing. 22582570. Retrieved from: https://www.proquest.com/openview/4b39ab5143062a2b1fa12c1e8f0f6279/1.pdf?pq-origsite=gscholar&cbl=18750&diss=y

Havaei, F., Astivia, O. L. O., & MacPhee, M. (2020). The impact of workplace violence on medical-surgical nurses' health outcome: A moderated mediation model of work environment conditions and burnout using secondary data. International Journal of Nursing Studies, 109, 103666. https://doi.org/10.1016/J.IJNURSTU.2020.103666

Al Sabei, S. D., Labrague, L. J., Miner Ross, A., Karkada, S., Albashayreh, A., Al Masroori, F., & Al Hashmi, N. (2020). Nursing Work Environment, Turnover Intention, Job Burnout, and Quality of Care: The Moderating Role of Job Satisfaction. Journal of Nursing Scholarship : An Official Publication of Sigma Theta Tau International Honor Society of Nursing, 52(1), 95–104. https://doi.org/10.1111/JNU.12528

Moisoglou, I., Yfantis, A., Galanis, P., Pispirigou, A., Chatzimargaritis, E., Theoxari, A., & Prezerakos, P. (2020). Nurses Work Environment and Patients' Quality of Care. International Journal of Caring Sciences, 13, 1–108. www.internationaljournalofcaringsciences.org

Sillero-Sillero, A., & Zabalegui, A. (2020). Analysis of the work environment and intention of perioperative nurses to quit work. Revista Latino-Americana de Enfermagem (RLAE), 28, 1–10. https://doi.org/10.1590/1518-8345.3239.3256

Kim, L. Y., Rose, D. E., Ganz, D. A., Giannitrapani, K. F., Yano, E. M., Rubenstein, L. V., & Stockdale, S. E. (2020). Elements of the healthy work environment associated with lower primary care nurse burnout. Nursing Outlook, 68(1), 14–25. https://doi.org/10.1016/J.OUTLOOK.2019.06.018

Kabakleh, Y., Zhang, J. P., Lv, M., Li, J., Yang, S., Swai, J., & Li, H. Y. (2020). Burnout and associated occupational stresses among Chinese nurses: A cross-sectional study in three hospitals. PLOS ONE, 15(9), e0238699. https://doi.org/10.1371/JOURNAL.PONE.0238699

Yuan, L., Yumeng, C., Chunfen, Z., & Jinbo, F. (2020). Analyzing the Impact of Practice Environment on Nurse Burnout Using Conventional and Multilevel Logistic Regression Models. Workplace health & safety, 68(7), 325–336. https://doi.org/10.1177/2165079919900796

| |
|---|
| Raso, R., Fitzpatrick, J. J., & Masick, K. (2020). Clinical Nurses' Perceptions of Authentic Nurse Leadership and Healthy Work Environment. JONA: The Journal of Nursing Administration, 50(9), 489–494. https://doi.org/10.1097/NNA.0000000000000921 |
| Wu, Y., Wang, J., Liu, J., Zheng, J., Liu, K., Baggs, J. G., Liu, X., & You, L. (2020). The impact of work environment on workplace violence, burnout and work attitudes for hospital nurses: A structural equation modelling analysis. Journal of Nursing Management, 28(3). https://doi.org/10.1111/JONM.12947 |
| Jameson, B. E., & Bowen, F. (2020). Use of the Worklife and Levels of Burnout Surveys to Assess the School Nurse Work Environment. The Journal of school nursing : the official publication of the National Association of School Nurses, 36(4), 272–282. https://doi.org/10.1177/1059840518813697 |
| Thomas, L., White, J., & Scanlon, K. (2020). Nurses' Perceptions of Their Practice Following a Redesign Initiative. Nursing Administration Quarterly, 44(4), E12–E24. https://doi.org/10.1097/NAQ.0000000000000437 |
| Paguio, J. T., Yu, D. S. F., & Su, J. J. (2020). Systematic review of interventions to improve nurses' work environments. Journal of Advanced Nursing, 76(10), 2471–2493. https://doi.org/10.1111/JAN.14462 |
| Gensimore, M. M., Maduro, R. S., Morgan, M. K., McGee, G. W., & Zimbro, K. S. (2020). The Effect of Nurse Practice Environment on Retention and Quality of Care via Burnout, Work Characteristics, and Resilience: A Moderated Mediation Model. The Journal of Nursing Administration, 50(10), 546–553. https://doi.org/10.1097/NNA.0000000000000932 |
| de Magalhães, A. M. M., de Freitas Cunha, D. R. M., de Moura, G. M. S. S., de Souza Urbanetto, J., Wegner, W., & de Oliveira, J. L. C. (2020). The environment of the nursing team's professional practice in inpatient units of a university hospital. Revista Gaucha de Enfermagem, 41. https://doi.org/10.1590/1983-1447.2020.20190460 |
| Maziero, E. C. S., Cruz, E. D. de A., Teixeira, F. F. R., Brandão, M. B., & Krainski, E. T. (2020). Associação entre condições de trabalho da enfermagem e ocorrência de eventos adversos em Unidades Intensivas neopediátricas. Revista Da Escola de Enfermagem Da USP, 54, e03623. https://doi.org/10.1590/S1980-220X2019017203623 |
| Poghosyan, L., Ghaffari, A., Liu, J., Jin, H., & Martsolf, G. (2021). State policy change and organizational response: Expansion of nurse practitioner scope of practice regulations in New York State. Nursing Outlook, 69(1), 74–83. doi: 10.1016/j.outlook.2020.08.007. |
| Johansen, M. L., Cordova, P. B., & Weaver, S. H. (2021). Exploration of the Meaning of Healthy Work Environment for Nurses. Nurse Leader, 19(4), 383–389. https://doi.org/10.1016/j.mnl.2020.06.011 |
| Donley, J. (2021). The Impact of Work Environment on Job Satisfaction: Pre-COVID Research to Inform the Future. Nurse Leader, 19(6), 585–589. https://doi.org/10.1016/j.mnl.2021.08.009 |

| |
|---|
| Manning, J., & Jones, N. (2021). Improving Healthy Work Environments Through Specialty Nursing Professional Development. Journal of Radiology Nursing, 40(3), 241–245. https://doi.org/10.1016/j.jradnu.2021.05.006 |
| Soheili, M., Taleghani, F., Jokar, F., Eghbali-Babadi, M., & Sharifi, M. (2021). Oncology Nurses' Needs Respecting Healthy Work Environment in Iran: A Descriptive Exploratory Study. Asia-Pacific Journal of Oncology Nursing, 8(2), 188–196. https://doi.org/10.4103/APJON.APJON_64_20 |
| Tosun, A., & Yildirim, A. (2021). Evaluation of Nurse Manager Practice Environment. International Journal of Caring Sciences, 14, 1437. www.internationaljournalofcaringsciences.org |
| de Oliveira Riboldi, C., Gasparino, R. C., Kreling, A., de Oliveira Júnior, N. J., da Silveira Barbosa, A., & de Magalhães, A. M. M. (2021). Environment of the professional Nursing practice in Latin American countries: a scoping review. Online Brazilian Journal of Nursing, 20(1), 3. https://doi.org/10.17665/1676-4285.20216473 |
| Alsufyani, A. M., Almalki, K. E., Alsufyani, Y. M., Aljuaid, S. M., Almutairi, A. M., Alsufyani, B. O., Alshahrani, A. S., Baker, O. G., & Aboshaiqah, A. (2021). Impact of work environment perceptions and communication satisfaction on the intention to quit: an empirical analysis of nurses in Saudi Arabia. PeerJ, 9. https://doi.org/10.7717/PEERJ.10949 |
| Lopes, R. P., Oliveira, R. M., Gomes, M. S. D. B., Santiago, J. C. D. S., Silva, R. C. R., & de Souza, F. L. (2021). Professional practice environment and nursing work stress in neonatal units. Revista Da Escola de Enfermagem Da USP, 55, e20200539. https://doi.org/10.1590/1980-220X-REEUSP-2020-0539 |
| Jawahir, S., Anuar, N. N. M., Abdullah, S. F. S., Silvernayagam, S., & Tan, E. H. (2021). Perception of nurses on the practice environment: experience from Malaysia. The Medical Journal of Malaysia, 76(1), 73–79. https://europepmc.org/article/med/33510113 |
| Sarıköse, S., & Göktepe, N. (2022). Effects of nurses' individual, professional and work environment characteristics on job performance. Journal of Clinical Nursing, 31(5–6), 633–641. https://doi.org/10.1111/jocn.15921 |
| Möller, G., de Oliveira, J. L., Dal Pai, D., Azzolin, K., & de Magalhães, A. M. (2021). Ambiente de prática de enfermagem em terapia intensiva e burnout profissional. Revista Da Escola de Enfermagem Da USP, 55, 1–8. https://doi.org/10.1590/1980-220X-REEUSP-2020-00409 |
| Abraham, C. M., Zheng, K., Norful, A. A., Ghaffari, A., Liu, J., & Poghosyan, L. (2021). Primary Care Practice Environment and Burnout Among Nurse Practitioners. Journal for Nurse Practitioners, 17(2), 157–162. https://doi.org/10.1016/j.nurpra.2020.11.009 |
| Moisoglou, I., Yfantis, A., Tsiouma, E., & Galanis, P. (2021). The work environment of haemodialysis nurses and its mediating role in burnout. Journal of Renal Care, 47(2), 133–140. https://doi.org/10.1111/JORC.12353 |

| |
|---|
| Kagan, I., Hendel, T., & Savitsky, B. (2021). Personal initiative and work environment as predictors of job satisfaction among nurses: cross-sectional study. BMC Nursing, 20(1), 1–10. https://doi.org/10.1186/s12912-021-00615-1 |
| Amaliyah, E., & Tukimin, S. (2021). The relationship between working environment and quality of nursing care: an integrative literature review. British Journal of Healthcare Management, 27(7), 194–200. https://doi.org/10.12968/bjhc.2020.0043 |
| Gurková, E., Bartoníčková, D., & Mikšová, Z. (2021). NURSING WORK ENVIRONMENT AND UNFINISHED NURSING CARE IN HOSPITAL SETTINGS - A SCOPING REVIEW. Central European Journal of Nursing & Midwifery, 12(3), 470–485. https://doi.org/10.15452/CEJNM.2021.12.0015 |
| Raso, R., & Fitzpatrick, J. (2021). How leadership matters: Clinical nurses' perceptions of leader behaviors affecting their work environment. Nursing Management, 52(10), 16–22. https://doi.org/10.1097/01.NUMA.0000792008.51038.f4 |
| Al Sabei, S. D., AbuAlRub, R., Labrague, L. J., Ali Burney, I., & Al-Rawajfah, O. (2021). The impact of perceived nurses' work environment, teamness, and staffing levels on nurse-reported adverse patient events in Oman. Nursing Forum, 56(4), 897–904. https://doi.org/10.1111/NUF.12639 |
| Ghasemi, R., Ghafourifard, M., Hassankhani, H., & Dehghannezhad, J. (2021). The Association of Work Environments and Nurse-Nurse Collaboration: A Multicenter Cross-Sectional Study. Nurse Media Journal of Nursing, 11(3), 370–379. https://doi.org/10.14710/nmjn.v11i3.41065 |
| Alenazy, F. S., Dettrick, Z., & Keogh, S. (2023). The relationship between practice environment, job satisfaction and intention to leave in critical care nurses. Nursing in Critical Care, 28(2), 167–176. https://doi.org/10.1111/NICC.12737 |
| Kassahun, C. W., Abate, A. T., Tezera, Z. B., Beshah, D. T., Agegnehu, C. D., Getnet, M. A., Abate, H. K., Yazew, B. G., & Alemu, M. T. (2022). Working environment of nurses in public referral hospitals of West Amhara, Ethiopia, 2021. BMC Nursing, 21(1), 1–8. https://doi.org/10.1186/S12912-022-00944-9/TABLES/3 |
| Jarrar, M., Al-Bsheish, M., Aldhmadi, B. K., Albaker, W., Meri, A., Dauwed, M., & Minai, M. S. (2021). Effect of Practice Environment on Nurse Reported Quality and Patient Safety: The Mediation Role of Person-Centeredness. Healthcare, 9(11). https://doi.org/10.3390/healthcare9111578 |
| Al Yahyaei, A., Hewison, A., Efstathiou, N., & Carrick-Sen, D. (2022). Nurses' intention to stay in the work environment in acute healthcare: a systematic review. Journal of Research in Nursing : JRN, 27(4), 374–397. https://doi.org/10.1177/17449871221080731 |
| Ulrich, B., Cassidy, L., Barden, C., Varn-Davis, N., & Delgado, S. A. (2022). National Nurse Work Environments - October 2021: A Status Report. Critical Care Nurse, 42(5), 58–70. https://doi.org/10.4037/CCN2022798 |
| Teixeira, G., Lucas, P., & Gaspar, F. (2022). International Portuguese Nurse Leaders' Insights for Multicultural Nursing. International journal of environmental research and public health, 19(19), 12144. https://doi.org/10.3390/ijerph191912144 |

Bruyneel, A., Bouckaert, N., Maertens de Noordhout, C., Detollenaere, J., Kohn, L., Pirson, M., Sermeus, W., & Van den Heede, K. (2023). Association of burnout and intention-to-leave the profession with work environment: A nationwide cross-sectional study among Belgian intensive care nurses after two years of pandemic. International journal of nursing studies, 137, 104385. https://doi.org/10.1016/j.ijnurstu.2022.104385

Prestia, A. S. (2022). Nurse Leader Cultivation of Rituals: Providing a Sense of Community and Connection. Nurse Leader, 20(4), 340–343. https://doi.org/10.1016/j.mnl.2022.01.006

Al Sabei, S. D., Al-Rawajfah, O., AbuAlRub, R., Labrague, L. J., & Burney, I. A. (2022). Nurses' job burnout and its association with work environment, empowerment and psychological stress during COVID-19 pandemic. International journal of nursing practice, 28(5), e13077. https://doi.org/10.1111/ijn.13077

Potrebny, T., Igland, J., Espehaug, B., Ciliska, D., & Graverholt, B. (2022). Individual and organizational features of a favorable work environment in nursing homes: a cross-sectional study. BMC Health Services Research, 22(1), 1–8. https://doi.org/10.1186/S12913-022-08608-9/TABLES/2

Molina Zavala, B. I., Zamora-Macorra, M., & Martínez Alcántara, S. (2022). Working Conditions and the Components of Burnout Among Nursing Staff in a Public Hospital in Mexico City. The journal of nursing research : JNR, 30(4), e219. https://doi.org/10.1097/jnr.0000000000000495

Pogue, C. A., Li, P., Swiger, P., Gillespie, G., Ivankova, N., & Patrician, P. A. (2022). Associations among the nursing work environment, nurse-reported workplace bullying, and patient outcomes. Nursing forum, 57(6), 1059–1068. https://doi.org/10.1111/nuf.12781

Poghosyan, L., Stein, J. H., Liu, J., Spetz, J., Osakwe, Z. T., & Martsolf, G. (2022). State-level scope of practice regulations for nurse practitioners impact work environments: Six state investigation. Research in Nursing & Health, 45(5), 516–524. https://doi.org/10.1002/NUR.22253

Poghosyan, L., Liu, J., Perloff, J., D’Aunno, T., Cato, K. D., Friedberg, M. W., & Martsolf, G. (2022). Primary Care Nurse Practitioner Work Environments and Hospitalizations and ED Use Among Chronically Ill Medicare Beneficiaries. Medical Care, 60(7), 496–503. https://doi.org/10.1097/MLR.0000000000001731

Patrician, P. A., Olds, D. M., Breckenridge-Sproat, S., Taylor-Clark, T., Swiger, P. A., & Loan, L. A. (2022). Comparing the Nurse Work Environment, Job Satisfaction, and Intent to Leave Among Military, Magnet ® , Magnet-Aspiring, and Non-Magnet Civilian Hospitals. JONA: The Journal of Nursing Administration, 52(6), 365–370. https://doi.org/10.1097/NNA.0000000000001164

Mabona, J. F., van Rooyen, D., & Ham-Baloyi, W. Ten. (2022). Best practice recommendations for healthy work environments for nurses: An integrative literature review. Health SA Gesondheid, 27(0), 11. https://doi.org/10.4102/HSAG.V27I0.1788

Borges, A. R. (2022). Ambiente de prática profissional e segurança do paciente pediátrico : implicações no enfrentamento da pandemia COVID-19. (dissertation). University of Porto Alegre

Carneiro, F. (2022). O Ambiente da Prática de Enfermagem e o desempenho das Unidades de Saúde Familiar num Agrupamento de Centros de Saúde. (dissertation). Nursing School of Lisbon

Vincelette, C., D'Aragon, F., Stevens, L. M., & Rochefort, C. M. (2023). The characteristics and factors associated with omitted nursing care in the intensive care unit: A cross-sectional study. Intensive and Critical Care Nursing, 75, 103343. https://doi.org/10.1016/J.ICCN.2022.103343

Rodríguez-García, M. C., Martos-López, I. M., Casas-López, G., Márquez-Hernández, V. V., Aguilera-Manrique, G., & Gutiérrez-Puertas, L. (2023). Exploring the relationship between midwives' work environment, women's safety culture, and intent to stay. Women and Birth : Journal of the Australian College of Midwives, 36(1), e10–e16. https://doi.org/10.1016/J.WOMBI.2022.04.002

Ribeiro, O. M. P. L., Cardoso, M. F., de Lima Trindade, L., da Rocha, C. G., Teles, P. J. F. C., Pereira, S., Coimbra, V., Ribeiro, M. P., Reis, A., da Conceição Alves Faria, A., da Silva, J. M. A. V., Leite, P., Barros, S., & Sousa, C. (2023). From the first to the fourth critical period of COVID-19: what has changed in nursing practice environments in hospital settings? BMC Nursing, 22(1), 1–12. https://doi.org/10.1186/S12912-023-01207-X/TABLES/5

Eva, G. F., Amo-Setién, F., César, L. C., Concepción, S. S., Roberto, M. M., Jesús, M. M., & Carmen, O. M. (2023). Effectiveness of intervention programs aimed at improving the nursing work environment: A systematic review. International Nursing Review. https://doi.org/10.1111/INR.12826
